# Ubiquitin-Conjugating Enzyme *OsUBC11* Affects the Development of Roots via Auxin Pathway

**DOI:** 10.1186/s12284-023-00626-3

**Published:** 2023-02-20

**Authors:** Yunfei Han, Chuanzhong Zhang, Hanjing Sha, Xiaojing Wang, Yue Yu, Jia Liu, Guangxin Zhao, Jingying Wang, Guankai Qiu, Xingjian Xu, Jun Fang

**Affiliations:** 1 State Key Laboratory of Black Soils Conservation and Utilization, Northeast Institute of Geography and Agroecology, Chinese Academy of Sciences, Harbin, China; 2 University of Chinese Academy of Sciences, Beijing, China; 3 Hinggan League Institute of Agricultural and Animal Husbandry Sciences, Hinggan League, 137400 Inner Mongolia China; 4 Northeast Agricultural University, Harbin, China

**Keywords:** Auxin, Root development, Ubiquitin-conjugating enzyme, Auxin transport, Ubiquitin degradation signal

## Abstract

Rice has 48 ubiquitin-conjugating enzymes, and the functions of most of these enzymes have not been elucidated. In the present study, a T-DNA insertional mutant named R164, which exhibited a significant decrease in the length of primary and lateral roots, was used as the experimental material to explore the potential function of *OsUBC11*. Analysis using the SEFA-PCR method showed that the T-DNA insertion was present in the promoter region of *OsUBC11* gene, which encodes ubiquitin-conjugating enzyme (E2), and activates its expression. Biochemical experiments showed that OsUBC11 is a lysine-48-linked ubiquitin chain-forming conjugase. *OsUBC11* overexpression lines showed the same root phenotypes. These results demonstrated that *OsUBC11* was involved in root development. Further analyses showed that the IAA content of R164 mutant and OE3 line were significantly lower compared with wild-type Zhonghua11. Application of exogenous NAA restored the length of lateral and primary roots in R164 and *OsUBC11* overexpression lines. Expression of the auxin synthesis regulating gene *OsYUCCA4/6/7/9*, the auxin transport gene *OsAUX1*, auxin/indole-3-acetic acid (Aux/IAA) family gene *OsIAA31*, auxin response factor *OsARF16* and root regulator key genes, including *OsWOX11, OsCRL1, OsCRL5* was significantly down-regulated in *OsUBC11* overexpressing plants. Collectively, these results indicate that *OsUBC11* modulates auxin signaling, ultimately affecting root development at the rice seedling stage.

**Supplementary Information:**

The online version contains supplementary material available at 10.1186/s12284-023-00626-3.

## Background

Root development is a vital process in plant growth. The functions of root include uptake of nutrients and water from soil, fixing in the soil to prevent logging, and serving as a major receptor interface between plants and various biotic and abiotic factors in the soil environment (Liu et al. 2005, 2009; Zhao et al. 2009; Kitomi et al. 2011). Therefore, exploring the molecules that regulate mechanisms associated with root development is essential in improving agricultural production and food security. The root system comprises primary root (PR), formed during embryo development, and crown roots (CR) which are formed after embryo development stage. Crown roots develop on non-root tissue such as the stems and tillering nodes (Bellini et al. 2014; Inukai et al. 2005), whereas lateral roots (LR) are formed on the surface of these roots. Development of these roots is modulated by various factors, such as phytohormones including auxin, ethylene, cytokinin, abscisic acid, and environmental stimuli (such as water and nutrients) (Gao et al. 2014; Liu et al. 2016a, b; Van et al. 2015; Huang et al. 2022). Although several phytohormones regulate root development, auxin is the main phytohormone that modulates development of roots (Overvoorde et al. 2010).

Auxin is involved in several plant growth and development processes, such as root and stem growth and development, vascular tissue formation, organ senescence and response to stress conditions (such as extreme temperatures, drought and flood, salt and alkali conditions). Root growth is modulated by the auxin pathway mainly through auxin biosynthesis, auxin transport and auxin signal transduction (Zhao et al. 2010). Overexpression of the auxin synthesis gene *OsYUCCA1* increases density of lateral root and the length of primary root (Yuko et al. 2007). Mutants of the auxin influx transport pivotal factor *OsAUX1*/*OsAUX3* and efflux carriers of the PIN family exhibit dysregulated root growth and density of lateral roots (Wang et al. 2009; Zhao et al. 2015a, b). T-DNA insertional mutant of the auxin respond factor *OsARF16* have longer root compared with the wild type. In addition to auxin, other auxin-related genes affect root development. For example, *OsWOX11* is a key gene involved in crown and root formation, which forms WOX11-ADA2-GCN5 complex and changes the chromatin status of target genes (Zhao et al. 2015a, b). Expression of downstream genes, implicated in auxin transport, cell composition and energy metabolism, is up-regulated in *oswox11* mutant. The *WOX11* gene regulates the development of roots through the interaction between cytokinin and auxin (Zhao et al. 2015a, b). *Ostdd1* gene encodes anthranilate synthase β-subunit, and plants with a mutation in this gene showed severe root development defects, and overexpression of *OsYUCCA1* reversed the defects (Sazuka et al. 2009). *AtUBC13A/B* encodes ubiquitin conjugation enzyme and the double-mutant affects root development by modulating auxin signaling (Wen et al. 2014).

The ubiquitination pathway is mainly involved in hormone signal transduction, flower development, photomorphogenesis and other biological processes (Moon et al. 2004; Sadanandom et al. 2012). The ubiquitin pathway mainly comprises ubiquitin-protein, E1 ubiquitin activator, E2 ubiquitin conjunction enzyme (abbreviated as E2 in this paper), E3 ubiquitin ligase and 26S protein degradation system. Approximately 6 E1s, 48 E2s, and 1300 E3s have been reported in rice (Bae and Kim 2014). The ubiquitin pathway involves tagging and degradation of substrate proteins through three enzymatic cascades, ultimately modulating transcriptional and post-translational activities in plants. The C-terminal glycine of the Ub molecule forms a bond with the ε-amino lysine residue of target proteins resulting in Ub enzymatic cascades (Wen et al. 2014). The lysine residue is linked through mono-ubiquitination or by forming a poly-ubiquitination chain. This poly-ubiquitination chain is formed through interaction with the lysine residues in Ub. Currently, K48-linked and K63-linked are the widely explored poly-ubiquitination types (Trempe et al. 2011). K48-linked poly-ubiquitination is a typical ubiquitination pathway involved in hormone signaling, pollen tube growth, pathogen defense, and the cell cycle (Smalle and Vierstra 2004). Studies have reported that K48-linked poly-ubiquitination is mainly associated with degradation-associated protein (Andrew et al. 2009). The SCF^TIR1/AFB2^ pathway is involved in degradation of Aux/IAA proteins, whereby auxin binds to TIR1/AFB2 to activate the SCF^TIR1/AFB2^ E3 complex resulting in ubiquitination of Aux/IAA proteins and their degradation through the 26S degradation pathway. Auxin response factor (ARF) is released, which enhances the auxin signaling (Maraschin et al. 2009). Previous findings indicate that K-63-linked poly-ubiquitination mainly plays several roles, such as restoration of DNA damage, kinase activation, protein synthesis, and regulation of root development (Jacobsen et al. 2009). Previous findings indicate that E2 contains a 150 aa conserved UBC domain, which is ubiquitous in eukaryotic cells. Studies report that 47 *OsUBCc* genes, with the exception of *OsUBC38,* have the conserved UBC domain (Liu et al. 2016a, b). UBC domain binds to the Ring domain of E3 to form a ubiquitin complex system, which mediates the downstream signaling. Studies have shown that E2 plays a vital role in plant stress resistance as well as plant growth and development (Wen et al. 2014). In Arabidopsis, *AtUBC13* is implicated in response to iron deficiency stress (Li et al. 2010). The *OsUBC6/8/9/22/25/43/45* gene family in rice modulates ABA stress (Zhiguo et al. 2015). *OsUBC1/2* is implicated in drought resistance (Joungsu et al. 2019), whereas *EL5* and *OsUBC5b* play roles in response to stress by inducing the ubiquitin/proteasome system (Hanae et al. 2007). These findings indicate a relationship between E3 and auxin levels and function. However, the correlation between OsUBCc and hormones has not been explored. Therefore, studies should be conducted to explore the mechanism of regulation of downstream auxin signaling by OsUBCc, and whether other pathways are implicated in regulating rice development.

A root development deficiency mutant was established and R164 was obtained from T-DNA insertional mutant bank to explore the association between E2 and auxin. Molecular and genetic analyses revealed that the mutant phenotype was associated with upregulation of a putative gene that encodes a ubiquitin conjunction enzyme, OsUBC11. In this study, a detailed analysis of *OsUBC11* was conducted and its role in modulating rice root development was explored.

## Results

### Phenotypes of R164 Mutant

**Fig. 1:**
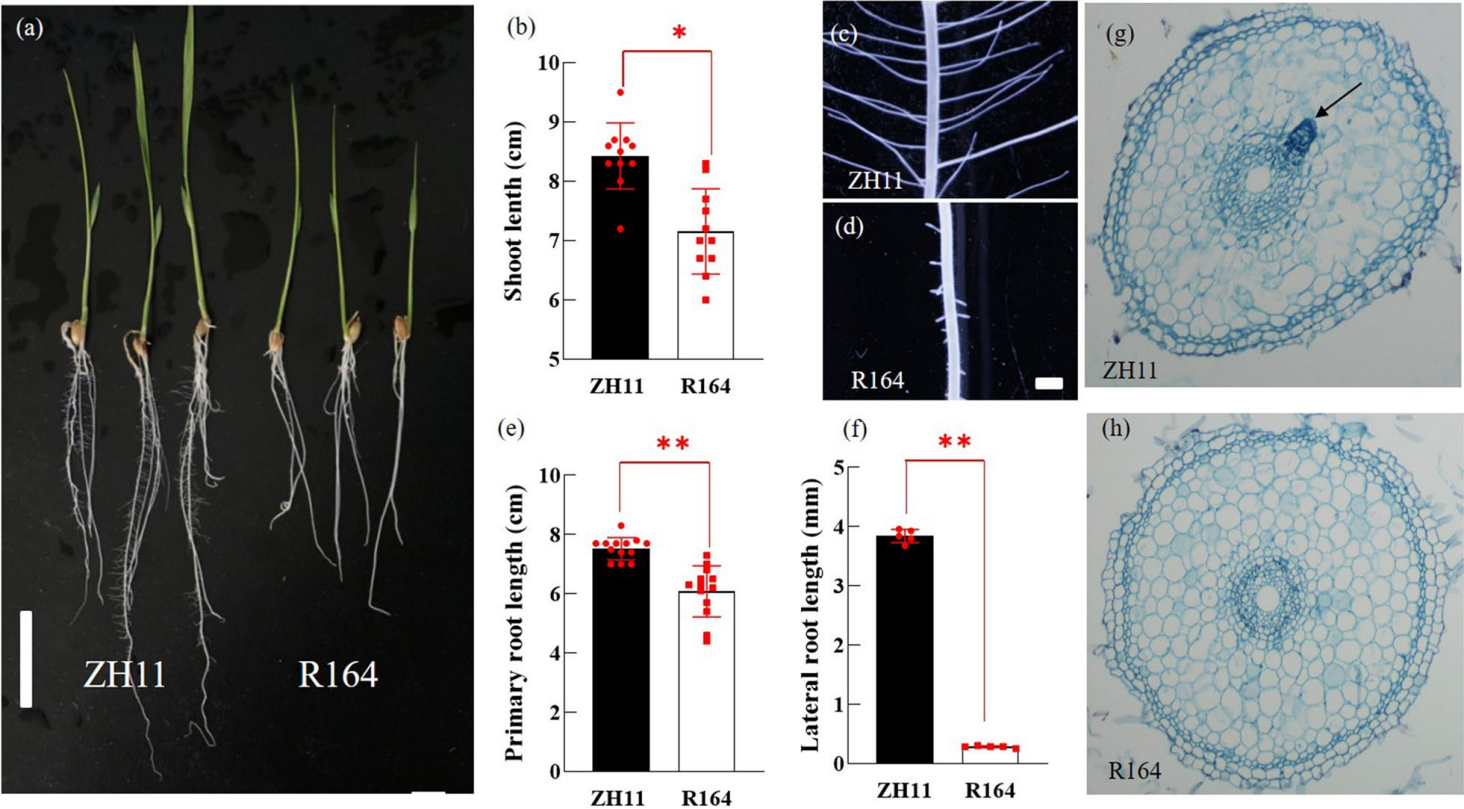
Phenotypes of the R164 mutant. **a** Root systems of wild-type (ZH11) and R164 seedlings at 7 DAG. Bar = 3 cm. **b** Determination of shoot length between ZH11 and R164 at 7 DAG. Red spot represented a duplicate value. One asterisk (*P < 0.05) represent significant differences between the WT and mutants. **c**, **d** The observation of microscopic examination of lateral roots of ZH11 and R164, respectively. Bar = 1 mm. **e** Determination of primary root length between ZH11 and R164 at 7 DAG. Red spot represented a duplicate value. Two asterisks (**P < 0.01) represent significant differences between the WT and mutants. **f** Determination of lateral root length between ZH11 and R164 at 7 DAG. Red spot represented a duplicate value. Two asterisks (**P < 0.01) represent significant differences between the WT and mutants. **g**, **h** Cross sections of the primary root of ZH11 (**g**) and R164 (**h**) mutant seedlings at 3 DAG, respectively. Black arrows indicate lateral root primordia

A root development deficiency mutant denoted as R164 was obtained from the T-DNA insertional mutant bank. The results showed that the primary and lateral roots had a lower development rate than the wild-type Zhonghua11 and the shoot length was affected at the seedling stage (Fig. 1a–f). Characterization of R164 mutant showed that the length of primary roots reduced by about 20% compared with WT plant (Fig. 1e) and the length of lateral root found was only 10% compared with WT by microscopic observation (Fig. 1c, d, f). Further analysis showed that the primary root of a 3-day-old WT presented a lateral root primordium, which was absent in the R164 mutant (Fig. 1g, h). In addition, R164 mutant exhibited several different phenotypic variations throughout the reproductive life, such as differences in plant height during the heading date stage compared with the WT (Additional file 1: Fig. S1c). The uppermost internode of the R164 mutant was shorter than that of the wild-type plant (Additional file 1: Fig. S1a, b). R164 mutant also showed shriveled pollen and increased number of aborted pollen grains (Additional file 1: Fig. S1d–f), as well as reduced seed setting rate and panicle length (Additional file 1: Fig. S1g–i).

### The Relative Expression of *OsUBC11* was Activated in R164 Mutant

**Fig. 2:**
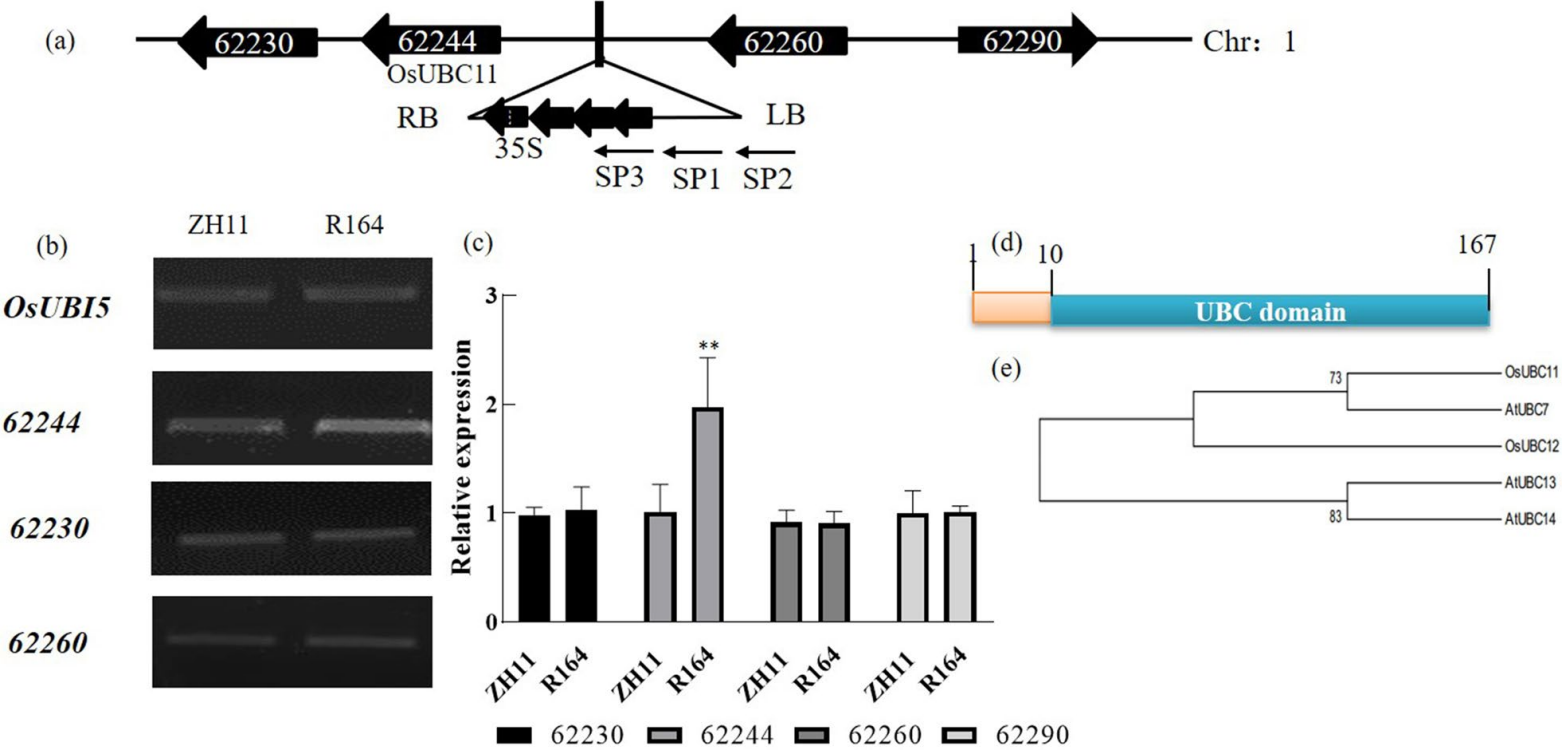
Identification of the T-DNA insert situation. **a** Schematic of the genomic region flanking the T-DNA insertion site in R164. 62230, 62244, 62260 and 62290 represent *LOC_Os01g62230*, *LOC_Os01g62244*, *LOC_Os01g62260* and *LOC_Os-01g62290* respectively; four arrow represent the four tandem copies of the *CaMV35S* enhancer near the T-DNA right border. LB, T-DNA left border. RB, T-DNA right border. The black arrows represent the P1, P2, and P3 primers used in find insert site. **b** Semiquantitative analysis of the insert site nearby genes, *OsUBI5* were amplified as control. **c** qRT-PCR analysis of the expression of *OsUBC11* between R164 mutant and WT Zhonghua11,*OsUBI5* transcripts were amplified as control. Red spot represented a duplicate value. Two asterisks (**P < 0.01) represent significant differences between the WT and mutants. **d** The *OsUBC11* protein domain. **e** Cladogram of *OsUBC11*

Self-formed adaptor PCR (SEFA-PCR) method was performed to confirm the T-DNA insertional site (Gao et al. 2014). The right border of T-DNA exhibited four 35S enhanced elements, which can activate the genes in the insertional site. Analysis showed that this T-DNA insertional site was present in Chr1 36023995 site (National Center for Biotechnology Information). Two genes were observed on the left and right border of this locus. The two significantly may affected genes were *LOC_01g62230* and *LOC_Os01g62244* on the right border and *LOC_Os01g62260* and *LOC_Os01g62290* on the left border (Fig. 2a). Expression of *LOC_Os01g62244* was significantly up-regulated, whereas semi-quantitative PCR analysis showed that the expression of the other three genes was not significantly different compared with WT (*OsUBI5* treated with reference gene) (Fig. 2b). *LOC_Os01g62244* gene showed 200% higher expression level compared with WT (*OsUBI5* treated with reference gene) (Fig. 2c). Analysis of rice database (https://rapdb.dna.affrc.go.jp/) showed that *LOC_Os01g62244* encodes ubiquitin conjugation enzyme (E2) named *OsUBC11*, which has a conserved UBCc domain (Fig. 2d). A total of 48 genes encode E2 in rice and are classified into 15 groups. The *OsUBC11* gene is classified in the V group (Bae and Kim 2014). Arabidopsis has three homologous genes (*AtUBC7*, *AtUBC13* and *AtUBC14*) (Fig. 2e). These genes may participate in response to stress conditions by modulating the plant endoplasmic reticulum (ER)- associated degradation (ERAD) pathway (Feng et al. 2020).

### The Phenotypes of *OsUBC11* Overexpression Lines Were Similar to R164

**Fig. 3:**
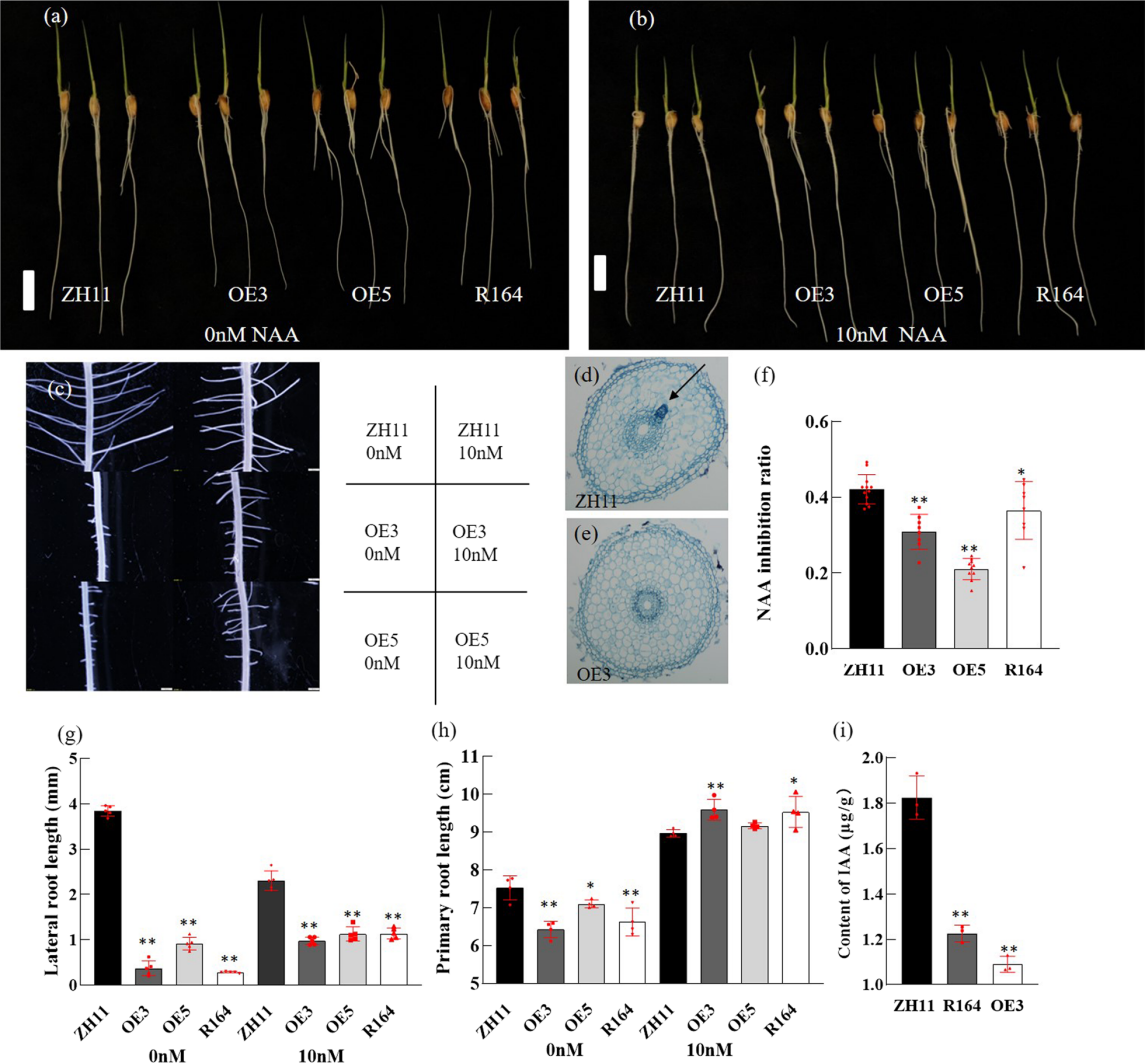
NAA treatment recovered the phenotype of *OsUBC11* overexpression lines. **a** The observation of 7 DAG seedling phenotype with 0 nM NAA treatment. Bar: 1.5 cm. **b** The observation of 7 DAG seedling phenotype with 10 nM NAA treatment. Bar: 2 cm. **c** The observation of 7 DAG seedling lateral roots phenotype in Microscopic examination of *OsUBC11* overexpression lines and ZH11.Bar: 0.5 mm. **d**, **e** Cross sections of the primary root of wild-type (**d**) and OE3 (**e**) seedlings at 3 DAG, respectively. Black arrows indicate lateral root primordia. **f** Determination of auxin sensitivity between *OsUBC11* overexpression lines, R164 and Zhonghua11, red spot represented a duplicate value. Two asterisks (**P < 0.01) and one asterisk (*P < 0.05) represent significant differences between the WT and mutants. **g** Determination of lateral root length between *OsUBC11* overexpression lines and ZH11, red spot represented a duplicate value. Two asterisks (**P < 0.01) and one asterisk (*P < 0.05) represent significant differences between the WT and mutants. **h** Determination of primary root length between ZH11 and *OsUBC11* overexpression lines. Red spot represented a duplicate value. Two asterisks (**P < 0.01) and one asterisk (*P < 0.05) represent significant differences between the WT and mutants. (i) Determination of auxin content in R164 mutant, OE3 and Zhonghua11. Red spot represented a duplicate value. Two asterisks (**P < 0.01) represent significant differences between the WT and mutants

The coding region of *OsUBC11* was cloned into the binary vector pCAMBIA2300-actin1-ocs to generate an *OsUBC11* overexpression vector in which *OsUBC11* was driven by the actin1 promoter, then transformed into Zhonghua11, to verify that overexpression of *OsUBC11* was responsible for the R164 phenotypes. Five independent T0 transgenic plants were identified in this study, with higher expression levels of lines 3 and 5 relative to the WT (Additional file 1: Fig. S2e). These two lines were selected for subsequent experiments (denoted as OE3 and OE5 in the subsequent sections). The *OsUBC11* overexpression plants and R164 mutant showed a slower rate of root development (Fig. 3a, c, g, h) and significantly shorter primary and lateral root length compared with the WT. The lateral root primordium was absent in OE3 at 3 DAG and in the R164 mutant (Fig. 3d, e). Auxin is associated with root development; thus, auxin concentration in R164, OE3 and WT plants was determined. The results showed that the auxin concentrations of R164 and OE3 were significantly lower than WT (Fig. 3i). Treatment with exogenous auxin increased the length of primary roots in the overexpression lines or the R164 mutant (Fig. 3b, h). Lateral root elongation in *OsUBC11* overexpression lines and R164 mutant was also recovered after treatment with 10 nmol NAA (Fig. 3c, g). These results indicate that the phenotype of root was associated with a decrease in auxin concentration. The rate of inhibition of the primary root of WT after treatment with NAA was significantly higher than that of *OsUBC11* overexpression lines (Fig. 3f). This finding implies that *OsUBC11* overexpression decreased the sensitivity of roots to auxin. *OsUBC11* overexpression plants exhibited similar aerial phenotypes as the R164 mutant, such as plant height, the length of the uppermost internode, shriveled pollen and the number of aborted pollen grains (Additional file 1: Fig. S2a–d, f, g). The results further indicate that *OsUBC11* modulated root, plant height and pollen development. The *osubc11* mutant was constructed by CRISPR technology. The T1 transgenic plants were harvested and sequencing was conducted. Three mutants, named *osubc11-1*, *osubc11-4* and *osubc11-9*, were identified (Additional file 1: Fig. S3a). The results did not show significant phenotype differences between *osubc11* mutants at the seedling stage and the wild-type (Additional file 1: Fig. S3b, c). We speculated that *OsUBC11* could have homologous gene, these genes had redundancy. The next step we would screened out the homologous gene and constructed the double mutant to observed the phenotype.

### The Expression of Auxin-Related Genes Were Altered in *OsUBC11* Overexpressing Lines

**Fig. 4:**
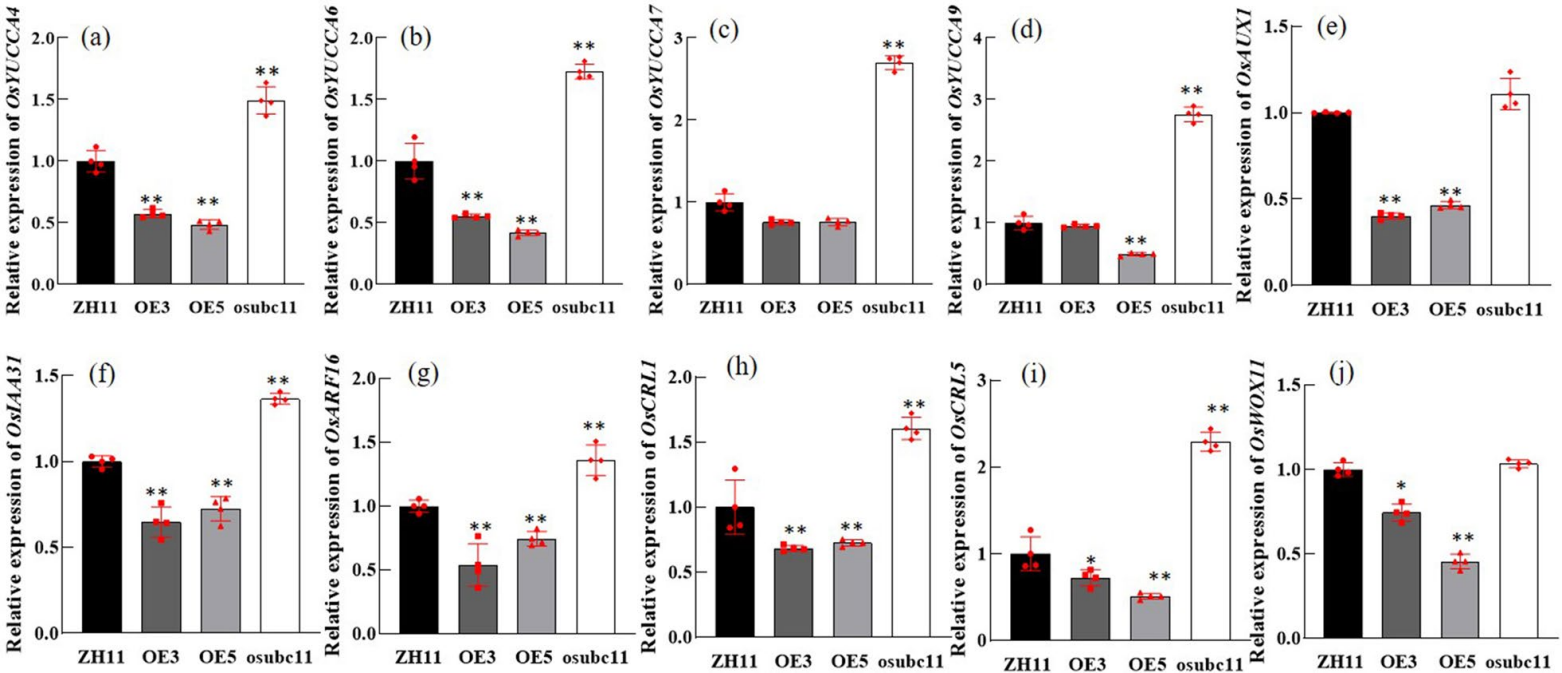
Expression relationship between auxin related genes and *OsUBC11*. **a** Relative expression of *OsYUCCA4* among different lines, compared with wild-type.* OsUBI5* transcripts were amplified as controls. Red spot represented a duplicate value. Two asterisks (**P < 0.01) represent significant differences between the WT and mutants. **b** Relative expression of *OsYUCCA6* among different lines, compared with wild-type.* OsUBI5* transcripts were amplified as controls. Red spot represented a duplicate value. Two asterisks (**P < 0.01) represent significant differences between the WT and mutants. **c** Relative expression of *OsYUCCA7* among different lines, compared with wild-type.* OsUBI5* transcripts were amplified as controls. Red spot represented a duplicate value. Two asterisks (**P < 0.01) represent significant differences between the WT and mutants. **d** Relative expression of *OsYUCCA9* among different lines, compared with wild-type.* OsUBI5* transcripts were amplified as controls. Red spot represented a duplicate value. Two asterisks (**P < 0.01) represent significant differences between the WT and mutants. **e** Relative expression of *OsAUX1* among different lines, compared with wild-type.* OsUBI5* transcripts were amplified as controls. Red spot represented a duplicate value. Two asterisks (**P < 0.01) represent significant differences between the WT and mutants. **f** Relative expression of *OsIAA31* among different lines, compared with wild-type. *OsUBI5* transcripts were amplified as controls. Red spot represented a duplicate value. Two asterisks (**P < 0.01) represent significant differences between the WT and mutants. **g** Relative expression of *OsARF16* among different lines, compared with wild-type.* OsUBI5* transcripts were amplified as controls. Red spot represented a duplicate value. Two asterisks (**P < 0.01) represent significant differences between the WT and mutants. **h** Relative expression of *OsCRL1* among different lines, compared with wild-type.* OsUBI5* transcripts were amplified as controls. Red spot represented a duplicate value. Two asterisks (**P < 0.01) represent significant differences between the WT and mutants. (i) Relative expression of *OsCRL5* among different lines, compared with wild-type.* OsUBI5* transcripts were amplified as controls. Red spot represented a duplicate value. Two asterisks (**P < 0.01) and one asterisk (*P < 0.05) represent significant differences between the WT and mutants. **j** Relative expression of *OsWOX11* among different lines, compared with wild-type.* OsUBI5* transcripts were amplified as controls. Red spot represented a duplicate value. Two asterisks (**P < 0.01) and one asterisk (*P < 0.05) represent significant differences between the WT and mutants

The expression profiles of genes implicated in auxin biosynthesis and degradation were explored to verify the association between *OsUBC11* and auxin levels. The expressions of auxin synthesis genes, *OsYUCCA4*, *OsYUCCA6*, *OsYUCCA7* and *OsYUCCA9* were down-regulated in *OsUBC11* overexpressing lines (Fig. 4a-d), but up-regulated in *osubc11* mutant lines (*osubc11-1* was used as the experimental material). The expression levels of *OsYUCCA1*, *OsYUCCA2* and auxin degradation genes *OsGH3.2* and *OsGH3.11* were not significantly different compared with wild type (Additional file 1: Fig. S4a–d). The results indicated that overexpression of *OsUBC11* inhibited auxin synthesis by partly reducing transcription levels of auxin synthesis genes, which was consistent with the decreased auxin concentrations observed in R164 and OE3 lines. Analysis was also conducted to determine the expression levels of the auxin transport genes, *OsPIN1*, *OsAUX1* and *OsAUX3* (Fig. 4e and Additional file 1: Fig. S4e–f). The findings showed that the expression of the auxin influx carrier encoded gene, *OsAUX1* was down-regulated in *OsUBC11* overexpressing lines compared with the WT. Expression of *OsAUX1* in *osubc11* mutant was up-regulated, indicating that *OsUBC11* repressed auxin transport. The levels of transcriptional repressors *OsIAA1* (Song et al. 2009), *OsIAA11* (Zhu et al. 2012), *OsIAA23* (Ni et al. 2011), *OsIAA31* (Zhang et al. 2019), and auxin response factor *OsARF12* (Qi et al. 2012) and *OsARF16* (Shen et al. 2013) were also evaluated (Additional file 1: Fig. S4g–l and Fig. 4f–g), to determine the effect of overexpression of *OsUBC11* gene on the development of crown roots and lateral roots. The results indicated that expression of *OsIAA31* and *OsARF16* was down-regulated in *OsUBC11* overexpression lines relative to the WT (Fig. 4f, g). These findings indicate that *OsUBC11* modulated auxin biosynthesis, auxin distribution and auxin signaling.

Further, the expression levels of crown root development marker genes in rice roots, including, *OsCRL1* (Inukai et al. 2005; Liu et al. 2005), *OsCRL5* (Kitomi et al. 2011), and *OsWOX11* (Zhao et al. 2015a, b) (Fig. 4h–j) were determined. Knockout or knockdown of these genes resulted in a shorter root phenotype consistent with the phenotypes of R164 and *OsUBC11* overexpression lines.

### OsUBC11 is a Lysine-48-Linked Ubiquitin Chain-Forming Conjugase

**Fig. 5:**
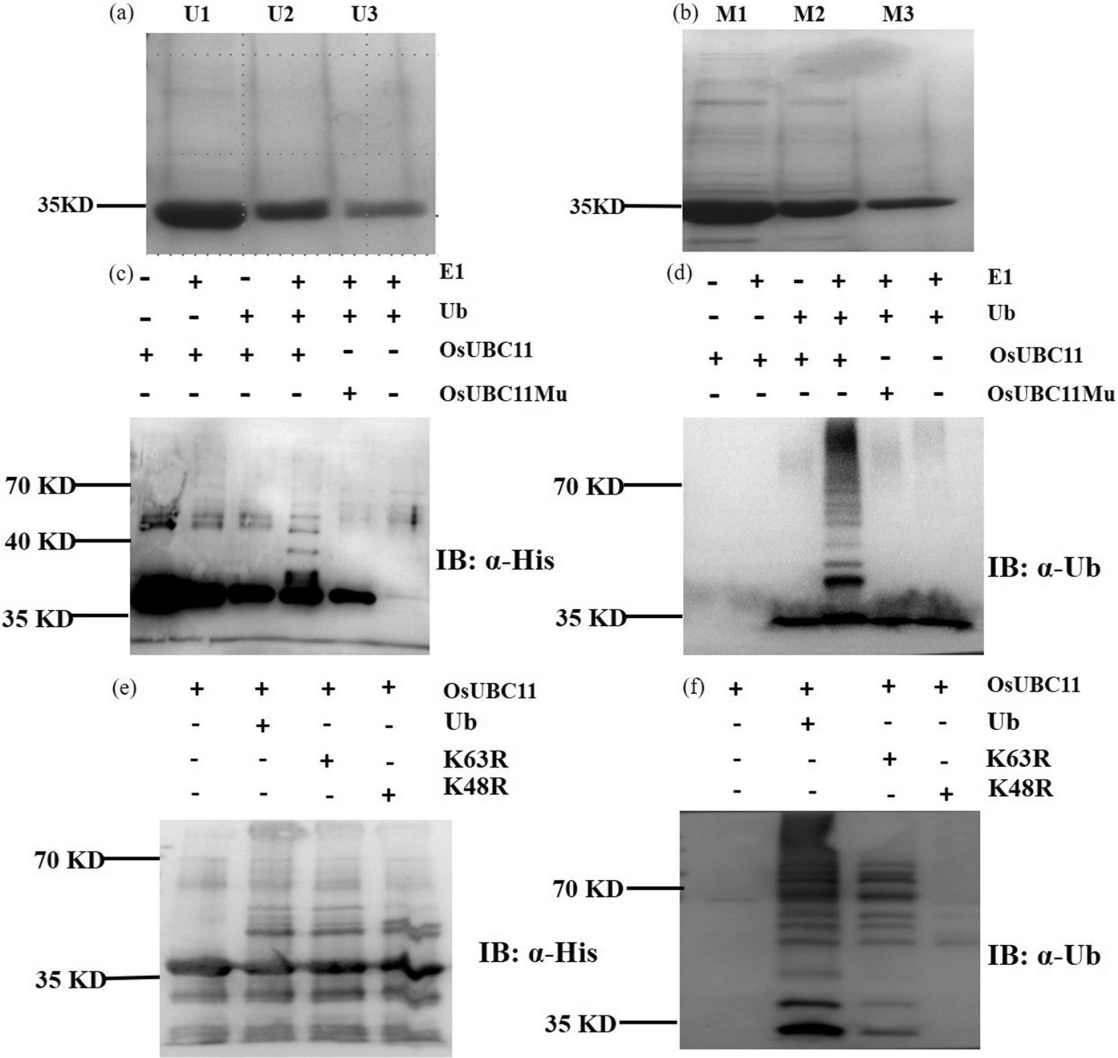
Determination of ubiquitin activity of OsUBC11. The recombinant protein purified result of OsUBC11-pET32b (**a**) and OsUBC11-Mu-pET32b (**b**) U1, U2, U3 represent elution times in OsUBC11, M1, M2, M3 represent elution times in OsUBC11 mutant protein. **c**, **d** Reaction of ubiquitin cascade of OsUBC11 or OsUBC11 mutant. Ubiquitinated proteins were detected by immunoblotting with anti-His (**c**) and anti-Ub (**d**). **e**–**f** Confirm the polyubiquitin-chain way of OsUBC11. Ubiquitinated proteins were detected by immunoblotting with anti-His (E) and anti-Ub (F)

OsUBC11-pET32b recombinant vector was constructed to explore the ubiquitin activity of OsUBC11. The conserved cysteine residue at position 92 of OsUBC11 protein sequence was mutated to alanine (UBC11-Mu) by point mutation technique. The recombinant proteins OsUBC11 and OsUBC11-Mu were expressed in large quantities through prokaryotic expression, tested and purified (Fig. 5a, b). Analysis of the ubiquitin cascade showed that OsUBC11 lost ubiquitination activity after mutation of cysteine at position 92 to alanine, indicating that OsUBC11 binds to the ubiquitination chain through the conserved cysteine residue (Fig. 5c, d). Subsequently, K48 and K63 residues of the ubiquitin chain were mutated to explore the mechanism of formation of polyubiquitin chain of OsUBC11. The results showed that the ubiquitin polymerization chain could not be formed after mutation of K48 residue (Fig. 5e–f), implying that OsUBC11 formed a polyubiquitination system through K48. Previous studies report that K48 ubiquitination is associated with protein degradation (Smalle and Vierstra 2004). These findings imply that OsUBC11 marks related substrate proteins through ubiquitination and degrades them through the ubiquitination pathway to regulate gene expression.

### Expression Pattern of *OsUBC11* and its Subcellular Localization

**Fig. 6:**
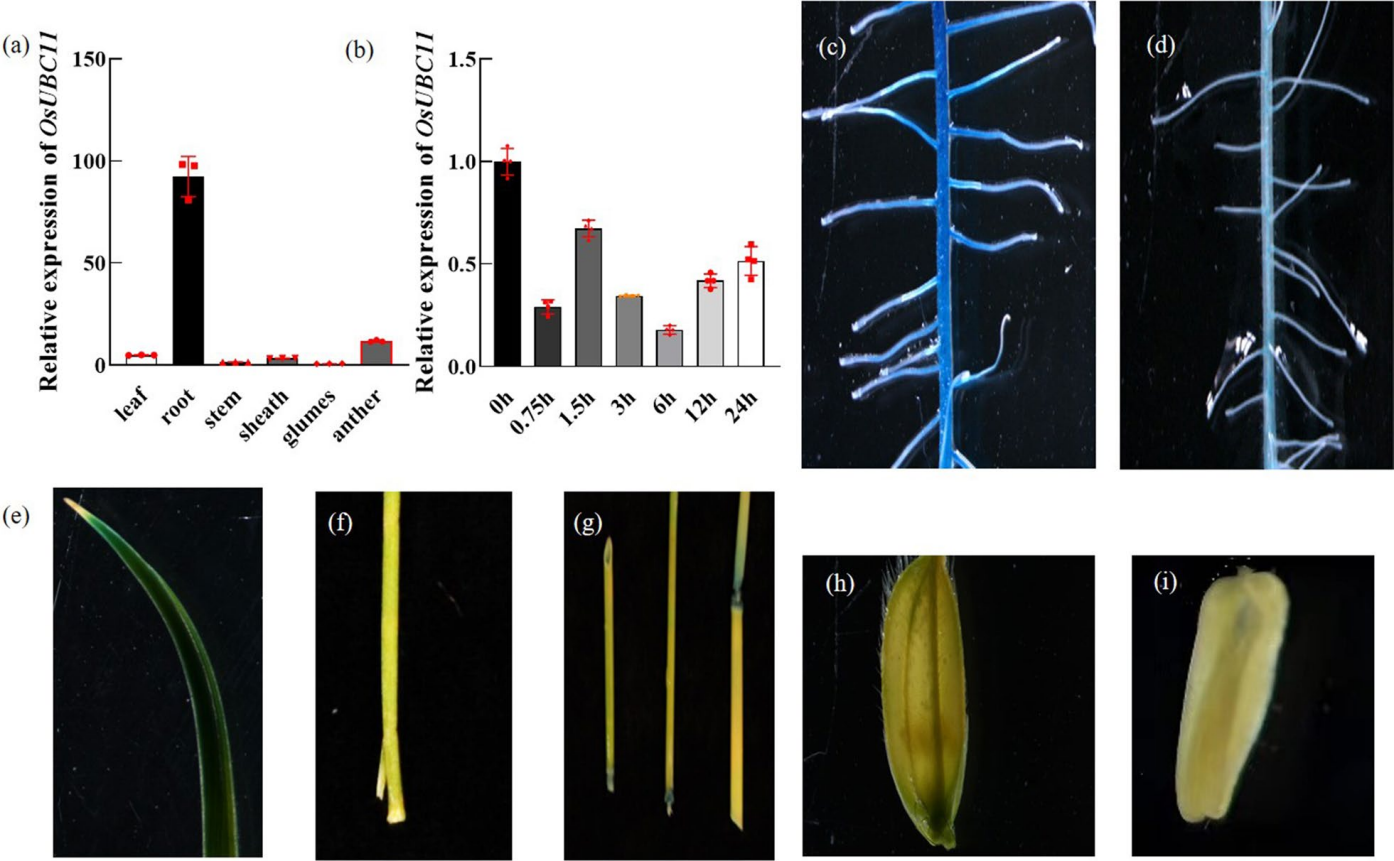
The expression analysis of *OsUBC11 *in different periods and tissues. **a** Expression pattern of *OsUBC11*,* OsUBI5* transcripts were amplified as controls, red spot represented a duplicate value. **b** *OsUBC11* express pattern in treatment with 1 μM NAA, *OsUBI5* transcripts were amplified as controls, red spot represented a duplicate value. **c** GUS staining in root at 7 DAG with no treatment. **d** GUS staining in root at 7 DAG treated with 1 μM NAA. **e** GUS staining in leaf at 7 DAG. **f** GUS staining in leaf sheath at reproductive stage. **g** GUS staining in stem and stem internode at reproductive stage. **h** GUS staining in glume at reproductive stage. **i** GUS staining in anther at reproductive stage

Total RNA was extracted from roots, stems, leaves of 1-week-old seedlings, sheath, glumes and rice anthers at the reproductive stage to explore the expression pattern of *OsUBC11*. The results showed that *OsUBC11* was mainly expressed in root but was also expressed at high levels in anthers (Fig. 6a). A 2 kb fragment upstream of the translation start site of *OsUBC11* was fused to the GUS reporter gene and transgenic plants were generated to evaluate the expression pattern of *OsUBC11* gene. Histochemical staining was conducted on samples obtained from T1 transgenic plants. Histochemical staining results of samples from 1-week-old transgenic lines and the heading stage tissues showed deep staining of root and internode, indicating consistent findings with the qRT-PCR results (Fig. 6c–i). These results may provide some understanding of the root development phenotype of R164 mutant. In addition, *OsUBC11* expression response to auxin was determined through treatment of plants with 1 µmol NAA treatment. qRT-PCR analysis revealed that the expression level of *OsUBC11* was significantly down-regulated within 0.75 h compared with the expression at 0 h (Fig. 6b). GUS staining of root samples showed auxin treatment reduced the degree of root staining (Fig. 6c, d), further indicating that the expression of *OsUBC11* was suppressed by auxin treatment.

GFP-*OsUBC11* recombinant plasmid was constructed and transformed to the protoplast of rice to evaluate *OsUBC11* subcellular localization. A vector expressing GFP alone was used as control. Microscopic analysis showed that *OsUBC11* gene was only expressed in cell nucleus (Additional file 1: Fig. S5), indicating that *OsUBC11* functions in the nucleus.

## Discussion

### *OsUBC11* Regulates Root Development and Plant Growth

Plant roots obtain water and nutrients from the soil. These resources are transported through the vascular system to other plant tissues and are essential for plant growth (Petricka et al. 2012; Smith et al. 2012). Several genes have been recently reported to regulate the growth of roots, most of which exhibit functional redundancy. The functions of these genes can be explored using root development mutants developed through the T-DNA insertion method (Gao et al. 2014). In the present study, a root development mutant, R164, was evaluated by SEFA-PCR method. The mutated gene encodes an ubiquitin conjunction enzyme (https://rapdb.dna.affrc.go.jp/) implicated in the ubiquitin cascade. Overexpression of *OsUBC11* inhibited development of primary roots and lateral roots. This finding implied that *OsUBC11* overexpression was the main cause of slow root development. Previous studies report that slow rate of root development is significantly correlated with auxin levels (Overvoorde et al. 2010). YUC (YUCCA encodes a flavin monooxygenase) and TAA/TAR (Tryptophan Aminotransferase of Arabidopsis) are gene families involved in the indole pyruvate pathway (IPA) associated with auxin synthesis (Zhao et al. 2012). Mutation of indole acetonitrile pathway associated genes, including *AtTGG4* and *AtTGG5*, causes defects in establishing an auxin gradient in the root tips resulting in suppressed root growth (Fu et al. 2016). The *AtUBC13A/B* mutation in the E2 gene family of *Arabidopsis thaliana* affects the auxin signaling factor, and down-regulates the expression of *YUC* family genes and *TAA* family genes, ultimately resulting in short roots (Wen et al. 2014). In this study, *OsUBC11* overexpression lines exhibited down-regulated expression of auxin synthesis regulator gene *OsYUCCA4/6/7/9,* although there is a degree of functional redundancy in these genes (Yuko et al. 2007). Triple- or quadruple- mutants exhibit root dysplasia (Stepanova et al. 2008; Cheng et al. 2006). The levels of auxin were significantly lower in the mutant compared with the WT, indicating that the function of *OsUBC11* is similar to that of the *Atubc13a/b* gene. This implies that *OsUBC11* down-regulates expression of *YUC4*, *YUC6*, *YUC7* and *YUC9* genes, which affects the synthesis of the auxin hormone, reduces the auxin content, and inhibits PR growth and LR development.

Auxin plays a central role in regulating root growth through modulation of auxin biosynthesis, transport, and signaling (Wang et al. 2009). Auxin transport and auxin signal transduction affect the distribution mode of auxins such as asymmetric distribution of auxins and intercellular transport (Zhao et al. 2015a, b; Qi et al. 2012). These effects ultimately determine the rate of cell division, cell differentiation, and cell expansion in plants (Friml 2021). The activities modulate auxin signaling intensities, thus affecting root development by altering auxin concentration in roots. Auxin influx carriers of the AUXIN1/LIKE AUX1 (AUX1/LAX) family and efflux carriers of the PIN-FORMED (PIN) family mediate polar auxin transport in plants and regulate root growth (Liu et al. 2016a, b). The *AUX1/LAX* family influx carriers in *Arabidopsis* comprises 4 genes, which encode four highly conserved transmembrane proteins denoted as *AtAUX1*, *AtLAX1*, *AtLAX2* and *AtLAX3* (Marchant et al. 2002; Swarup et al. 2004; Yang et al. 2006; Péret et al. 2012). Previous studies report that *AtAUX1* and *AtLAX3* genes modulate lateral root development (Marchant et al. 2002; Swarup et al. 2004). The homolog of *AtAUX1* gene, *OsAUX1* has a similar function, and its mutants are associated with reduced auxin transport. The homolog and mutants dysregulate the expression of cell cycle genes implicated in mobilizing auxin to the target root tissue to enhance elongation of roots (Huang et al. 2022), ultimately affecting development of lateral roots (Zhao et al. 2015a, b). *OsAUX3* functions as a negative regulator resulting in a decrease in rice Al-tolerance in roots (Wang et al. 2018). Inhibition of *OsPIN1* expression increases the tiller number and decreases crown root number. Overexpression of *OsPIN1* causes significant changes in the root-shoot ratio, implying that *OsPIN1* plays an important role in plant type establishment and development of adventitious roots in rice (Xu et al. 2005). *OsPIN2* overexpression changes the number of adventitious roots and lateral root density in rice (Chen et al. 2012). In this current study, expression of *OsAUX1* was repressed in OE3 and OE5 lines, whereas the expression levels of *OsPIN1* and *OsAUX3* were not significantly different compared with the WT, indicating that *OsUBC11* may be the upstream gene of *OsAUX1*.

The key components of the auxin signaling pathway include an E3 ligase complex, SCF^TIR1^, downstream proteins comprising two gene families, auxin response factor (ARF) and auxin/indole acetic acid (AUX/IAAs) (Guilfoyle and Hagen 2007). Auxin signal through the TIR receptor promotes degradation of AUX/indole acetic acid (IAA) proteins, which induces *ARF7/19* to regulate the expression of a series of downstream genes implicated in LR development (Pérez-Torres et al. 2008). For instance, SOR1-OsIAA26 complex acts downstream of *OsTIR1*/*AFB2*-*OsIAA9* cascade to regulate the root growth of rice seedlings (Hui et al. 2018). *OsIAA1* overexpression enhances root development (Song et al. 2009). *Osiaa11* mutant inhibits development of lateral roots (Zhu et al. 2012). *OsARF12* and *OsARF25* and auxin response factors and their knockout decrease primary root length in rice (Qi et al. 2012). The phenotype of *OsARF16* is similar to *OsARF12* phenotype and *OsPIN1b*, *OsPIN4* and *OsPIN9* are the downstream genes, which regulate auxin distribution, ultimately affecting root growth (Shen et al. 2013). The findings of the present study showed that overexpression of *OsUBC11* gene down-regulated expression of the auxin signal repressor gene *OsIAA31* (Zhang et al. 2019) and the auxin response factor *OsARF16* (Shen et al. 2013). *OsAUX1* and *OsARF16* genes may play a synergistic role in regulating the development of root hair (Jia et al. 2022). *OsIAA31* is associated with disease resistance and is mainly expressed in roots with a similar expression pattern as *OsUBC11* gene (Ayako et al. 2006; Zhang et al. 2019). A recent study showed that E2 modulates stress resistance and acts in response to other hormones, such as the hormone encoded by *AtUBC27*, thus positively regulating ABA signaling and response to drought (Pan et al. 2020). Most E2 are affected by at least two hormones (Zhiguo et al. 2015). The homologous of *OsUBC11* in *Arabidopsis* are *AtUBC7*, *AtUBC13* and *AtUBC14*, representing single, double, or triple mutants, which do not exhibit the phenotype presented by *OsUBC11* overexpressed lines. Notably, plants with the three mutants or triple mutant of *AtUBC7*, *AtUBC13* and *AtUBC14* respond to stress conditions and ABA in mutation dependent manner. These genes may be part of the plant endoplasmic reticulum (ER)- associated degradation (ERAD) pathway. ERAD removes misfolded proteins to maintain protein homeostasis (Feng et al. 2020). These findings imply that *OsUBC11* may respond to other hormones and is implicated in ERAD pathway through the K48-linked pathway, thus, it degrades associated proteins, which ultimately affects plant development. Further studies should be conducted to explore whether other hormones affect the expression profile of *OsUBC11* and the response of *OsUBC11* expression to abiotic stress.

In addition to root development, R164 mutant and *OsUBC11* overexpression lines were associated with aerial growth phenotypes, such as shortened top internode, shrinkage of anthers, and increased number of abortive pollen (Additional file 1: Figs. S1 and S2). GUS staining results were consistent with real-time PCR results (Fig. 6). Expression pattern analysis showed that *OsUBC11* gene was mainly expressed in roots (Fig. 6a). These results indicate that *OsUBC11* plays a critical role in rice development, especially in regulation of root development.

### *OsUBC11* Down-Regulates Expression of Genes Involved in Root Development

Our previous findings showed that *OsUBC11* overexpression affects root development, thus further analysis was conducted to explore whether *OsUBC11* could modulate expression of genes implicated in root growth. The results showed that expression of these genes was down-regulated in the plants overexpressing *OsUBC11* gene. *OsWOX11* gene is important for root development (Zhao et al. 2009). Loss-of-function mutation or down-regulation of *OsWOX11* gene results in reduced number and decreased growth rate of crown roots. In this study, the expression level of *OsWOX11* was lower in *OsUBC11* overexpression lines relative to the WT (Fig. 4j). WOX11-ADA2-GCN5 is a ternary complex in rice crown root formation. WOX11 plays a key role in crown root formation by modulating the cytokinin pathway (Zhao et al. 2015a, b). WOX11 interacts with ADA2 to recruit acetylase GCN5 by binding to specific downstream target genes. GCN5 acetylates histones at target gene loci and ultimately promotes expression of downstream genes implicated in auxin transport, cell composition and energy metabolism by changing the chromatin status of target genes (Zhou et al. 2017). Notably, down-regulation of *OsWOX11* expression was associated with low expression levels of *OsIAA31* and *OsIAA11* genes. *OsIAA11* gene regulates root development (Zhu et al. 2012; Jing et al. 2015). Overexpression of *OsWOX11* up-regulates expression of *OsIAA31* and *OsIAA11* genes, implying that *OsUBC11* inhibits the expression of *OsWOX11*, which further inhibits the expression of *OsIAA31* and *OsIAA11* genes. Previous findings demonstrated that the root development phenotypes of plants overexpressing *OsYUC* are dependent on the presence of *WOX11,* indicating that *WOX11* is a potential downstream gene for *OsYUC* genes (Zhang et al. 2018).

The expression level of *OsCRL1* and *OsCRL5* in this study was lower in *OsUBC11* overexpression lines compared with the WT. *OsCRL1* is downstream gene of AUX/IAA and ARF-mediated pathway and is regulated by *OsARF16* (Inukai et al. 2005; Liu et al. 2005). *OsCRL5* is a member of auxin signaling cascade and promotes root development, thus the mutant did not exhibit initiation of crown root primordia. *OsARF1* binds to the promoter of *OsCRL5* and further modulates the cytokinin signaling pathway through type-A response (ARRS) regulators (Kitomi et al. 2011). These results indicate that overexpression of *OsUBC11* inhibits these genes and further inhibits the growth of roots at the seedling stage.

## Conclusion

**Fig. 7:**
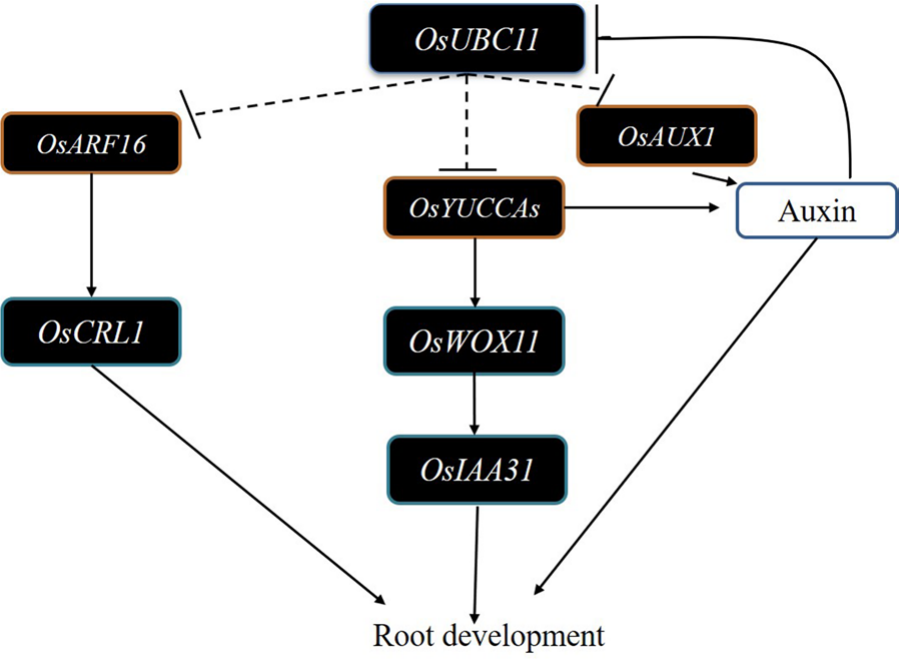
Model depicting the possible mode of *OsUBC11*. Overexpress *OsUBC11* could repress the expression of auxin biosynthesis genes, reduced the content of auxin, and further influenced the expression of root development gene *OsWOX11* and *OsIAA31.* At the same time, auxin intracellular transport gene *OsAUX1* and auxin signal genes *OsARF16* also be restrained if *OsUBC11* overexpression. Further regulate root development genes, *OsCRL1* and *OsCRL5*, totally retarded the growth of root. In addition, exogenous auxin can also inhibit the expression of *OsUBC11*

*OsUBC11* overexpression reduces the concentration of auxin and inhibits root development by modulating expression of genes involved in auxin synthesis and transport. The present findings show that administration of exogenous auxin can down-regulate expression of *OsUBC11* gene (Fig. 7).

## Materials and Methods

### Plant material and Growth Conditions

The R164 T-DNA insertional mutants and the wild type Zhonghua11 were stored in our laboratory. Seeds of R164 and WT were surfaced-sterilized and sowed in 1/2 strength MS medium, the 7-day-old seedlings were washed off the remnant medium, observing and statistics the length of shoot and root. For phenotype complement experiment, various seeds of *OsUBC11* were immersed in water at 37 °C for 24 h, then transformed hydroponic box, was floating on Kimura nutrient solution in a growth chamber with a 14 h light:10 h dark photoperiod at 28 °C for 7 days. Auxin treatment was NAA supplemented in distilled water, distilled water was treated as control. The observation of root and flower structure are in stereomicroscope (Olympus SZX16).

To observe the phenotype of reproductive stage, the overexpression line of *OsUBC11*, the CRISPER mutant of *OsUBC11* and wild type Zhonghua11 were grown in artificial shading outdoors in Harbin, 10 h light/14 h darkness.

### Identification of R164 T-DNA Insertion Mutant

The insert site was confirmed by Self-Formed Adaptor PCR method (Wang et al. 2007). T-DNA right border three primers were listed in Additional file 2: Table S1, Genomic DNA was extracted from leaves of R164 mutant using the CTAB method. By agarose gel electrophoresis found about 5 kb band, sequenced blast by NCBI (www.ncbi.nlm.nih.gov). The expression levels of *OsUBC11* in R164 were determined by semiquantitative PCR and Real time PCR. Semiquantitative PCR prime and real-time PCR prime were amplified as a control treatment, UBI5 (Os01g0328400) was chosen as internal control. All primes in this chapter were listed in Additional file 2: Table S1.

### Vector Constructions and Phenotype Observation

Vector construction was by using ClonExpress® technology (Vazyme Biotech Co., Ltd), or Gateway® technology (Thermo Fisher Scientific-CN).

To constructed the *OsUBC11* overexpression lines, a 510-bp fragment of *OsUBC11* CDS region was amplified from the cDNA. The restriction sites (Xma I and Pst I) were incorporated into the primers to facilitate cloned into the pCAMBIA2300-actin1 to form overexpression recombinant vector.

To constructed the *osubc11* crisper mutant, we designed the crisper target spot primer, primer listed in Additional file 2: Table S1, the target sequences was ligated in sgRNA, and subsequently inserted into CRISPR/Cas9 vector pYLCRISPR/Cas9Pubi‐H (Ma et al. 2015).

To constructed the *OsUBC11*-GUS recombinant vector, a 2000-bp fragment of upstream *OsUBC11* encoding region was amplified from genomic DNA of Zhonghua11, recombined with intermediate vector pQB-V3, then with the technology of LR reaction, take fragment recombined into pHGWFS7.0 plasmid.

Above recombined plasmid were transferred to *Agrobacterium* tumefaciens strain EHA105 and then transformed into wild-type Zhonghua11 as described previously (Karimi 2015). All of the primers used to generate the above-mentioned constructs are listed in Additional file 2: Table S1.

To constructed the *OsUBC11*-GFP recombined vector, a 510-bp fragment of *OsUBC11* CDS region was amplified, the method of the constructed was the same as *OsUBC11*-GUS. Then transform into protoplast of rice for further observation (The vector is pH7WGF2.0).

### The extracted of Protein and Ubiquitin Cascade Experiment

To confirm the ubiquitin activity of OsUBC11, the OsUBC11-His and site mutant protein-His all constructed by pET-32b vector, the proteins were purified by His-Bind resign (EMD Milillipore Corp, USA), the reagent and ubiquitin cascade experiment and K48/63 polyubiquitin confirm experiment is supported by YOUBI biology. Separated by 10% SDS‐PAGE, transferred to Immobilon®-P transfer membrane (Merck Millipore, IPVH00010), and detected by immunoblot analysis using anti-His (Abmart) and ubiquitin antibody Mab (Beytone), respectively.

### Measurement of Auxin and Sensitivity Analysis to Auxin

IAA concentration from roots of 7-day-old seedlings in WT and R164 plants was measured by High-performance liquid chromatography method, each sample was set three repetition.

The equation of sensitivity analysis to auxin is (the length of primary root in 0 nM NAA-the length of primary root in 10 nM NAA)/the length of primary root in 10 nM NAA. Experiments were repeated at least three times.

### GUS Staining

GUS staining of *OsUBC11*-GUS lines were harvested at different growth stages was performed using phosphate buffer with 2 mM X-Gluc, vacuum pump to drain, then immersed in these buffers for 48 h. After staining, the samples were observed under an Olympus SZX16 stereo microscope and photographed with a digital camera (Cannon 720S).

### Subcellular Localization of *OsUBC11*

To examine the subcellular localization of *OsUBC11*, the coding sequence of *OsUBC11* was fused in frame to of pH7WGF2.0. The *OsUBC11*-GFP fusion construct and empty GFP vector control were transfected into protoplast of rice were observed with a confocal laser scanning microscope (Leica TCSSP5).

### Assay of Real-Time PCR

Entire root of WT and other *OsUBC11* transgenic lines at 7-day-old were used for isolation total RNA (Takara 9109), reverse transcriptase was chosen ReverTra Ace qPCR RT Kit (TOYOBO, FSQ-301), Real-Time PCR system was conducted on LightCycler® 96 System (Roche) using TransStart® Top Green qPCR SuperMix(TransGene,AQ131). The amplification of *UBI5 *(*Os01g0328400*) was chosen as an internal control, and the relative expression of genes was calculated via the 2^−ΔΔCT^ method. Other alternative genes primer are listed in Additional file 2: Table S1. Experiments were repeated at least three times.

### Statistical Analysis

Data were analysed by GraphPad Prism 8, every single piece of data are shown as a black spot in the figure.

### Accession Numbers

The sequence data of this article can be found in the Rice Genome Annotation Project Database and Resource (http://rice.plantbiology.msu.edu) under the following accession numbers:*OsUBC11*(LOC_Os01g62244), *UBI5*(Os01g0328400), *OsGH3.2*(LOC_Os01g55940), *OsGH3.11*(LOC_Os07g47490), *OsYUCCA1*(LOC_Os01g45760), *OsYUCCA2*(LOC_Os05g45240), *OsYUCCA4*(LOC_Os01g12490), *OsYUCCA6*(LOC_Os07g25540), *OsYUCCA7*(LOC_Os04g03980), *OsYUCCA9*(LOC_Os01g16714), *OsAUX1*(LOC_Os01g63770), *OsAUX3*(LOC_Os05g37470), *OsPIN1*(LOC_Os04g02830), *OsIAA1*(LOC_Os01g08320), *OsIAA11*(LOC_Os03g43400), *OsIAA19*(LOC_Os05g48590), *OsIAA23*(LOC_Os06g39590), *OsIAA31*(LOC_Os12g40900), *OsARF1*(LOC_Os11g32110), *OsARF12*(LOC_Os04g57610), *OsARF16*(LOC_Os06g09660), *OsCRL1*(LOC_Os03g05510), *OsCRL5*(LOC_Os07g03250) and *OsWOX11*(LOC_Os07g48560).

## Supplementary Information

**Additional file 1**. **Fig S1**: Phenotype observation of R164 and ZH11. **Fig S2**: Phenotype observation of OsUBC11 overexpression lines and ZH11. **Fig S3**: Phenotype observation of osubc11 mutant. **Fig S4**: Determination of auxin relative genes. **Fig S5**: Subcellular location of OsUBC11.

**Additional file 2**. **Table S1**: Primers used in this study.

## Data Availability

The datasets used and/or analysed during the current study are available from the corresponding author on reasonable request.

## Abbreviations

NAA: Naphthylacetic acid
ZH11: Zhonghua11
OE3: *OsUBC11* Overexpression line 3
OE5: *OsUBC11* Overexpression line 5
E1: Ubiquitin activator
E2: Ubiquitin conjunction enzyme
E3: E3 ubiquitin ligase
GUS: β-Glucuronidase
qRT-PCR: Quantitative real-time PCR
GFP: Green fluorescent protein
DAG: Days after germination
SEFA-PCR: Self-formed adaptor PCR
