# Supplementary material for: Ubiquitin-Conjugating Enzyme OsUBC11 Affects the Development of Roots via Auxin Pathway
Source: Rice (N Y). 2023 Feb 20;16:9. doi: 10.1186/s12284-023-00626-3 (PMC9941415; doi:10.1186/s12284-023-00626-3)
Supplement: Supplementary file 1 — Additional file 1. Fig S1: Phenotype observation of R164 and ZH11. Fig S2: Phenotype observation of OsUBC11 overexpression lines and ZH11. Fig S3: Phenotype observation of osubc11 mutant. Fig S4: Determination of auxin relative genes. Fig S5: Subcellular location of OsUBC11. [file 12284_2023_626_MOESM1_ESM.docx]

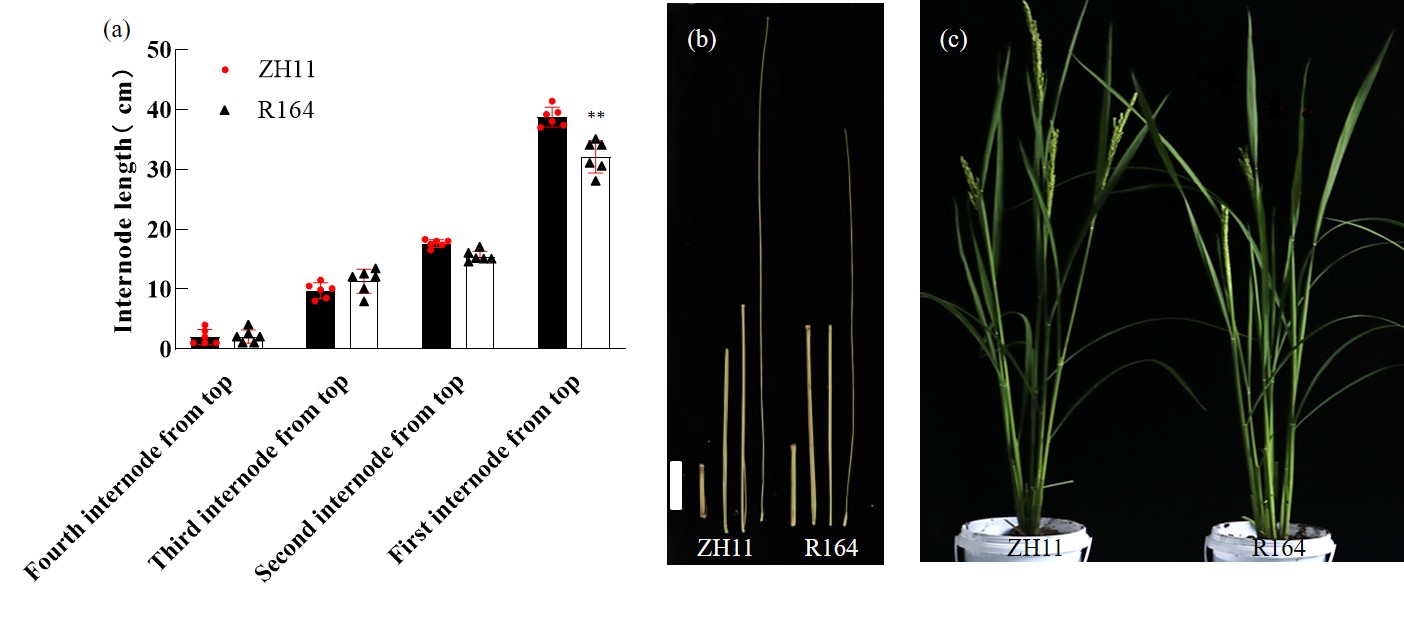


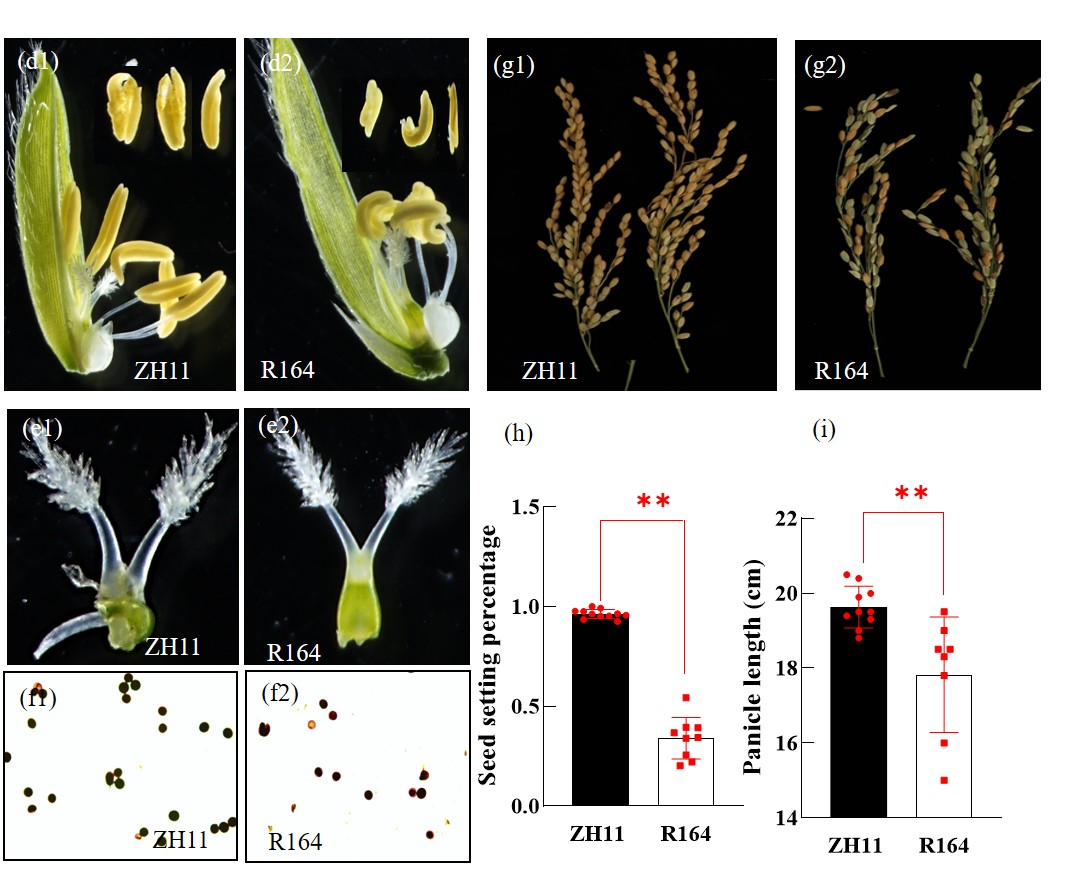


**Figure S1 Phenotype observation of R164 and Zhonghua 11**

Phenotype observation of R164 and Zhonghua 11. **(a)** Determination of the length of internode between R164 and Zhonghua11,from the left to right, are the fourth internode from top, the third internode from top, the second internode from top and the first internode from top, respectively. Red spot represented a duplicate value. Two asterisks (**, P < 0.01) represent significant differences between the WT and mutants. **(b)** The observation of internode, from the left to right, are the fourth internode from top, the third internode from top, the second internode from top and the first internode from top, respectively. And the first group of internode are wild-type Zhonghua11, the second group of internode are R164.Bar：1cm **(c)** The observation of plant length between Zhonghua11 and R164. **(d)** The structure of flower and glume of Zhonghua11 and R164. **(e)** The observation of pistil between Zhonghua11 and R164. **(f)** The observation of pollen between Zhonghua11 and R164. **(g)** The observation of panicle between Zhonghua11 and R164. **(h)** Determination of the seed setting percentage of Zhonghua11 and R164. Red spot represented a duplicate value. Two asterisks (**, P < 0.01) represent significant differences between the WT and mutants.. **(i)** Determination of the length of panicle between Zhonghua11 and R164. Red spot represented a duplicate value. Two asterisks (**, P < 0.01) represent significant differences between the WT and mutants.


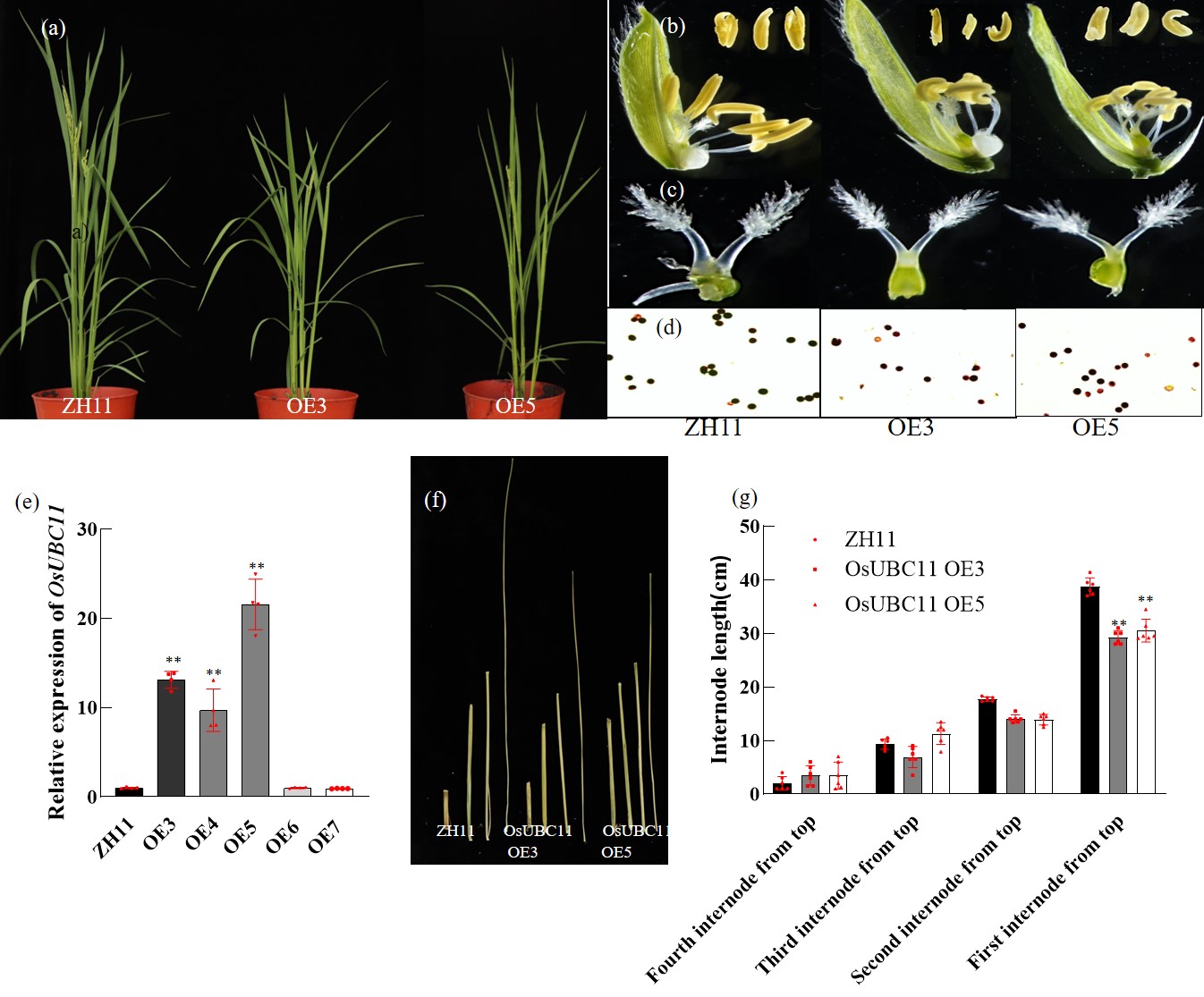


**Figure S2. Phenotype observation of OsUBC11 overexpression lines and Zhonghua 11**

Phenotype observation of OsUBC11 overexpression lines and Zhonghua 11. **(a)** The observation of plant length between Zhonghua11 and R164. **(b)** The structure of flower and glume of Zhonghua11 and OsUBC11 overexpression lines. **(c)** The observation of pistil between Zhonghua11 and OsUBC11 overexpression lines. **(d)** The observation of pollen between Zhonghua11 and OsUBC11 overexpression lines. **(e)** Determination of the relative expression of OsUBC11 between different lines, Red spot represented a duplicate value. Two asterisks (**, P < 0.01) represent significant differences between the WT and mutants. **(f)** The observation of internode, from the left to right, are the fourth internode from top, the third internode from top, the second internode from top and the first internode from top, respectively. And the first group of internodes are wild-type Zhonghua11, the second group of internodes are OE3 line, the third group of internodes are OE5 line.Bar:1cm. **(g)** Determination of the length of internode between OsUBC11 overexpression lines and Zhonghua11, from the left to right, are the fourth internode from top, the third internode from top, the second internode from top and the first internode from top, respectively, Red spot represented a duplicate value. Two asterisks (**, P < 0.01) represent significant differences between the WT and mutants.

**
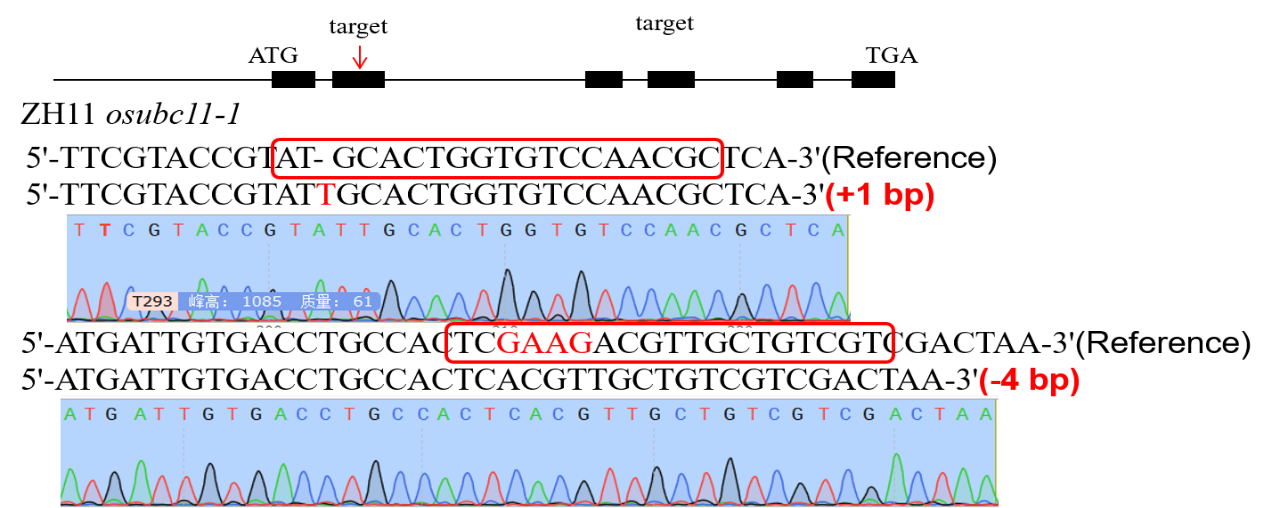

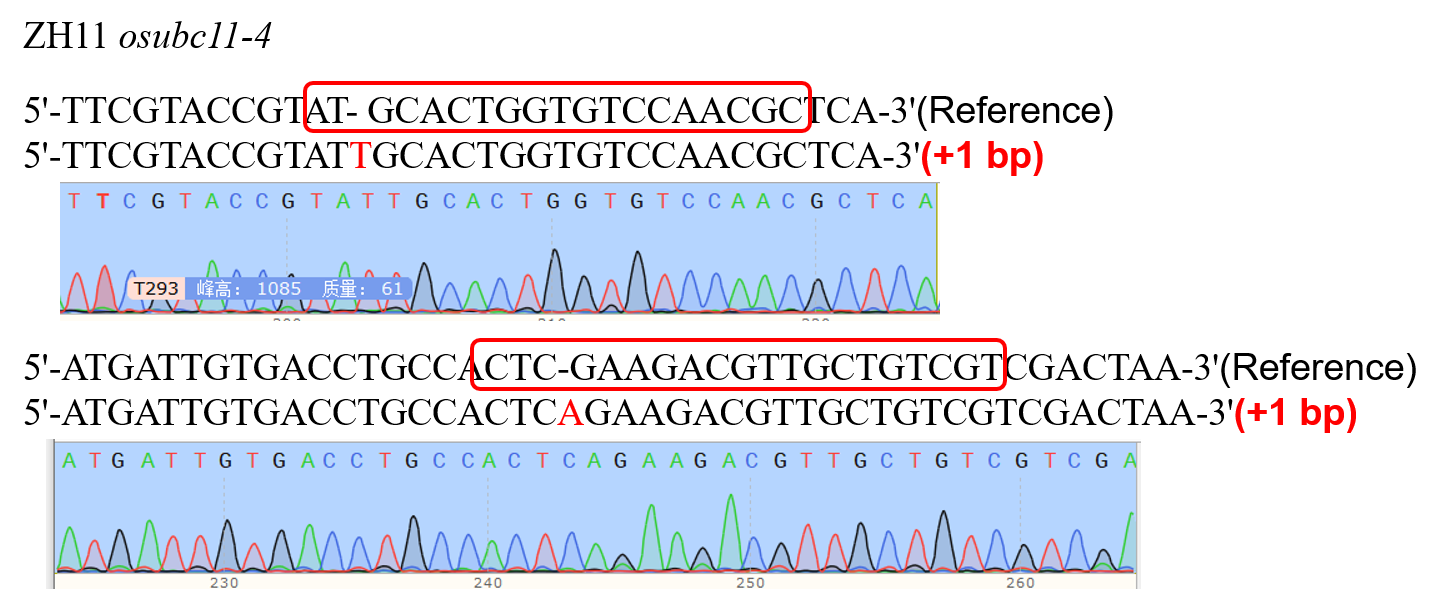

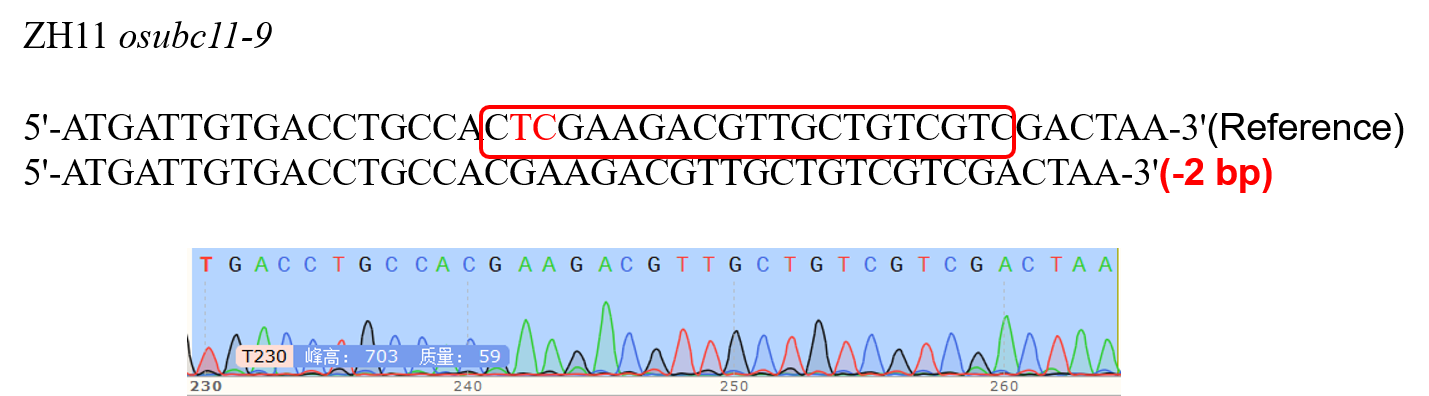
**

**
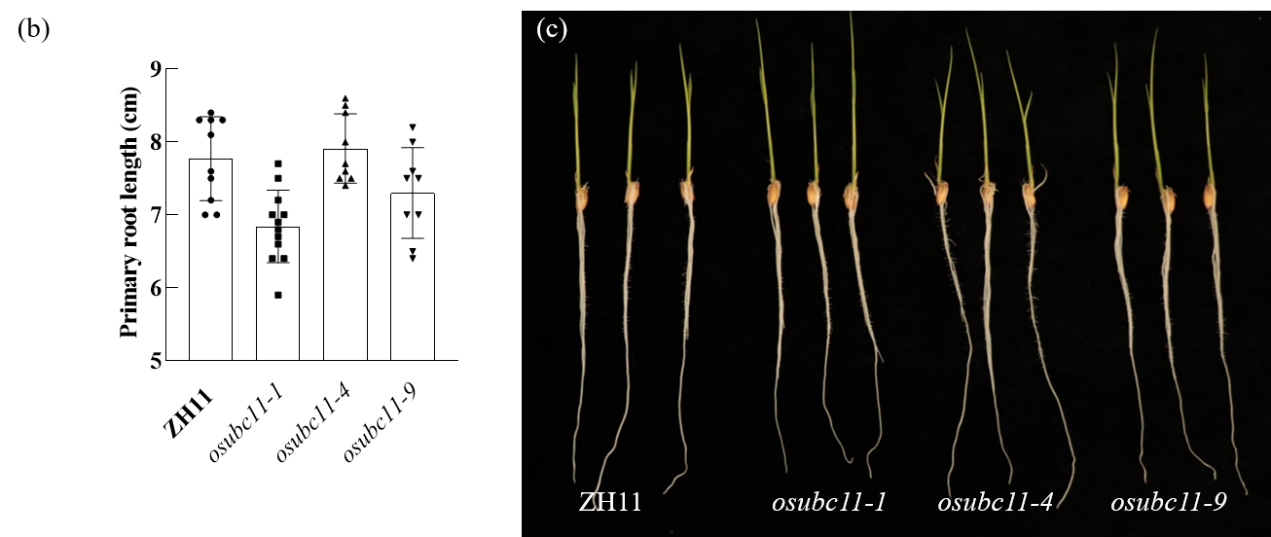
**

**Figure S3.** **Phenotype observation of *osubc11* mutant**

Phenotype observation of *osubc11* mutants. **(a)** The mutant site of *osubc11.* **(b)** Determination of primary root between Zhonghua11 and *ubc11* mutant. **(c)**The observation of root length between Zhonghua11 and *osubc11* mutants at 7-day-seedling. Bar:1cm.

**
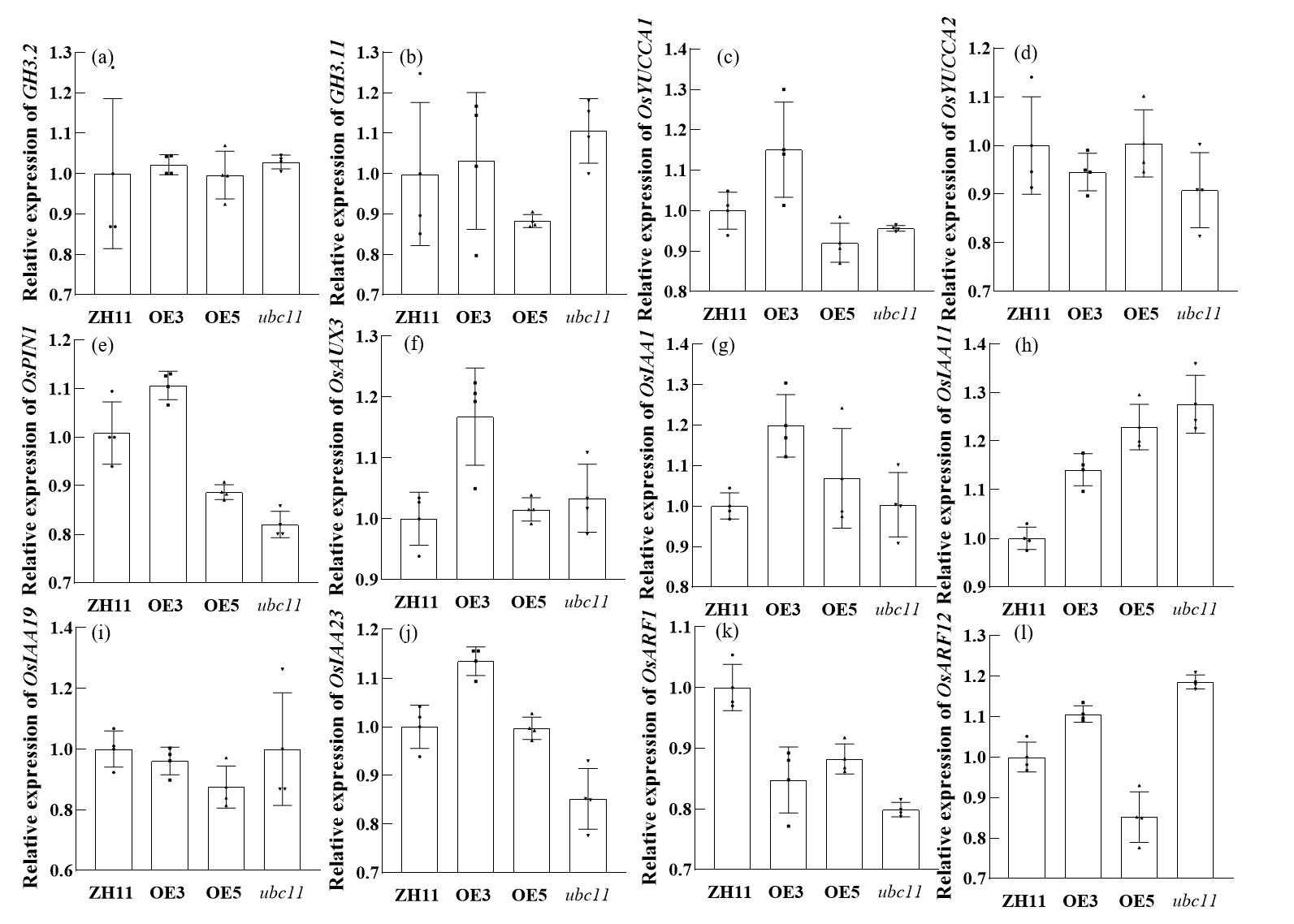
**

**Figure S4 Determination of auxin relative genes**

Determination of auxin relative genes. **(a-d)** Relative expression of auxin synthesis and degradation genes among different lines, compared with wild-type. *OsUBI5* transcripts were amplified as controls. Black spot represented a duplicate value. **(e-f)** Relative expression of auxin transport genes among different lines, compared with wild-type. *OsUBI5* transcripts were amplified as controls. Black spot represented a duplicate value. **(g-l)** Relative expression of auxin signal genes among different lines, compared with wild-type. *OsUBI5* transcripts were amplified as controls. Black spot represented a duplicate


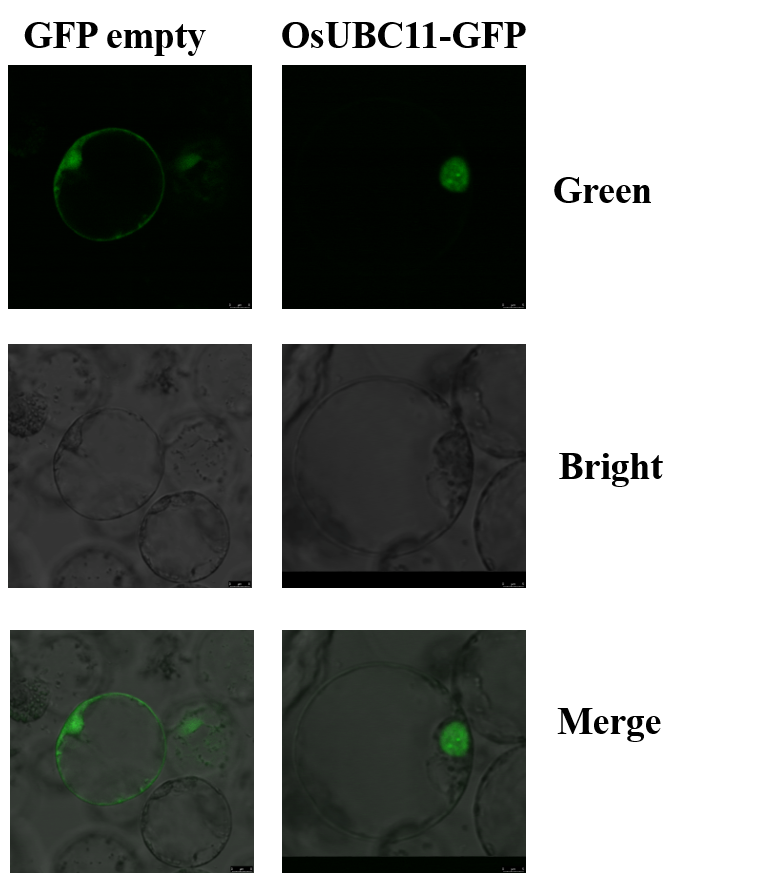


**Figure S5 Subcellular localization of O*sUBC11***
