# Supplementary material for: Ubiquitin-Conjugating Enzyme OsUBC11 Affects the Development of Roots via Auxin Pathway
Source: Rice (N Y). 2023 Feb 20;16:9. doi: 10.1186/s12284-023-00626-3 (PMC9941415; doi:10.1186/s12284-023-00626-3)
Supplement: Supplementary file 2 — Additional file 2. Table S1: Primers used in this study. [file 12284_2023_626_MOESM2_ESM.docx]

**Table S1** Primers used in this study.

| **Primer** | **Sequence (5’ to 3’)** | **Primer** | **Sequence (5’ to 3’)** | **used for** |
| --- | --- | --- | --- | --- |
| OsUBC11-U3F | GGCACGACAGCAACGTCTTCGAG | OsUBC11-U3R | AAACCTCGAAGACGTTGCTGTCG | **Vector construction** |
| OsUBC11-U6AF | GCCGCGTTGGACACCAGTGCATA | OsUBC11-U6AR | AAACTATGCACTGGTGTCCAACG |  |
| OsUBC11-OEF | CCCGGG ATGGCGGCTACCATCAGCCA | OsUBC11-OER | CTGCAGGCCCAAGCCACACAAATACA |  |
| OsUBC11-GUSF | GATATCCGCCTACATCGTCCATACCA | OsUBC11-GUSR | GATATCGAGATCGGCGTGGCTAGG |  |
| OsUBC11-GFPF | TCCCCCCGGGATGGCGGCTACCATCAGCCA | OsUBC11-GFPR | GCTCTAGATCAGAGCATTTCTTGTGATT |  |
| OsUBC11-32BF | GAATTCGATGGCGGCTACCATCAGCCA | OsUBC11-32BR | GTCGACTCAGAGCATTTCTTGTGATT |  |
| NPTII-F | TGCTCGCTCGATGCGATGTT | NPTII-R | CTCTGATGCCGCCGTGTTCC | **Genotyping** |
| Semi-62244F | GGTTCTCGGCTGGGTTAGT | Semi-62244R | GCTCTCAACCGTATGCACTG | **Semi-Quantitati-ve PCR** |
| Semi-62230F | ATCTCCAGCGAATTCCCGAT | Semi-62230R | TGTGGGAAATCTAAGAGCGC |  |
| Semi-62260F | CCACCACTCCATCACAGTCT | Semi-62260R | TGTTGTACATGTTGCGGCAG |  |
| Semi-62290F | AGGAAAGGCTAGTGGAGTGG | Semi-62290R | GGATTCGGCCCTTCCTTCTA |  |
| Semi-UBI5F | CCTCGGACACCATCGACAACGTG | Semi-UBI5R | CGCCCCCAAAGAACAGGAGCCTA |  |
| qRT-OsUBC11F | GGTTCTCGGCTGGGTTAGT | qRT-OsUBC11R | AATGCATACACGTCCATCCG | **qRT-PCR** |
| qRT-YUUCA1F | GAAGGTGTTGGTCGTGGGAT | qRT-YUUCA1R | CGATGCCGAACGTGGATAGA |  |
| qRT-YUUCA2F | TATGGCGGGTGAGGACTACA | qRT-YUUCA2R | GTCGGCTGACCTAGCATCTC |  |
| qRT-YUUCA4F | TGGCCTGTACGCTGTTGGTTTCT | qRT-YUUCA4R | CCATTCCATGTCAGTTGTTCTCA |  |
| qRT-YUUCA6F | GTCAGGGCTCTACTCTGTTGGCT | qRT-YUUCA6R | ATCTTGTGGTGGCGGTTTGGT |  |
| qRT-YUUCA7F | AGGAGGCGACGAAGCCAAC | qRT-YUUCA7R | TCAACAACGAATTTAACCAAGGG |  |
| qRT-YUCCA9F | CAACAATGGCTCGGCCTAGAAAG | qRT-YUCCA9R | CAAAGACCACCCAAGGGCAAGT |  |
| qRT-GH3.2F | TACATCCCCACGCTCAAGTT | qRT-GH3.2R | GCTCGAAGTAGCCCATGTTG |  |
| qRT-GH3.11F | CATTGCCCAACCCAGAGCTA | qRT-GH3.11R | AGCCATGTTTGGGAGGATGG |  |
| qRT-AUX1F | ACTACATCAACGACCGGCTG | qRT-AUX1R | CAGCACCAGCTTGGTTGGAC |  |
| qRT-AUX3F | TTGGAATTTTGCAGGTGGCG | qRT-AUX3R | ACCACTGGATGACGTGGTTC |  |
| qRT-PIN1F | AGTCTCGGTCTGTTCATGGC | qRT-PIN1R | ACTCCTTGGCGAAGACGAAG |  |
| qRT-PIN2F | CAACACCTACTCCAGCCTC | qRT-PIN2R | TGGACCAGTCAAGAACCTC |  |
| qRT-IAA1F | TCACCATCCGGAAGTTCGCC | qRT-IAA1R | GCTGGCAAGTTTCCACAAACA |  |
| qRT-IAA11F | GATGACCTGGAGTACGTGCC | qRT-IAA11R | AAGGCCAATGGCTTCAGACC |  |
| qRT-IAA19F | AGGATGCTGACTGGATGCTT | qRT-IAA19R | TTCCAGTGATCTCGGAGCCA |  |
| qRT-IAA23F | TGATCGATGAGCCAAATTGG | qRT-IAA23R | AGCAGGGAGCACACCTGAAT |  |
| qRT-IAA31F | CGGAGCTCCATACCTGAGGA | qRT-IAA31R | CTTGTTGCTCCTAGGCCTCT |  |
| qRT-ARF1F | GGTGTCTCCCCTACCTTGTC | qRT-ARF1R | CTTCAACAACCTTGGACGCA |  |
| qRT-ARF12F | TGTCTTTTGGGGGCTCGGTTT | qRT-ARF12R | AACCTTCACAAATGTTCTGGTT |  |
| qRT-ARF16F | GACCCCAACCAGCTCTATCG | qRT-ARF16R | TCCCAAAGGGTAGCAGAGAG |  |
| qRT-WOX11F | AGCAGCTCTACTACTCGTGTC | qRT-WOX11R | ATCTCAGATCGATCGGTCCC |  |
| qRT-CRL1F | ATGACGGGATTTGGATCGC | qRT-CRL1R | CTTGCTCGTGGCAGAAGTAT |  |
| qRT-CRL5F | CCCTTCCACACACATCAACT | qRT-CRL5R | CTCCTTAAGTGAGCCACATACTC |  |
| qRT-UBI5F | ACCACTTCGACCGCCACTACT | qRT-UBI5R | ACGCCTAAGCCTGCTGGTT |  |
| BD-UBC11F | GAATTCATGGCGGCTACCATCAGCCA | BD-UBC11R | CTGCAGTCAGAGCATTTCTTGTGATT | **Protein interaction** |
| AD-RBX1AF | GAATTCATGGACAAGGGCGACGTCGC | AD-RBX1AR | CTCGAGCTAAAGGAACAGCAGATCGG |  |
| AD-RBX1BF | GAATTCATGTCGGCCATGGAGACCGA | AD-RBX1BR | CTCGAGCTAGTGCCCATATTTCTGAA |  |
| UBC11-nLUCF | GGTACCATGGCGGCTACCATCAGCCA | UBC11-nLUCR | GTCGACTCAGAGCATTTCTTGTGATT |  |
| RBX1A-cLUCF | GGTACCATGGACAAGGGCGACGTCG | RBX1A-cLUCR | GTCGACCTAGTGACCATACTTCTGGAACTCCC |  |
| SP1 | AGGCATCGATCGTGAAGTTTCTCATC | SP2 | CATTTGGACGTGAATGTAGACACGTCG | **SEFA-PCR** |
| SP3 | GCTTTCGCCTATAAANNNNNNNNATCG |  |  |  |
